# Motivations to exercise in young men following a residential weight loss programme conducted in National Service - a mixed methods study

**DOI:** 10.1186/s12889-021-10373-z

**Published:** 2021-02-17

**Authors:** Alexander Wilhelm Gorny, Mui Cheng Low, Andrew Arjun Sayampanathan, Farah Shiraz, Falk Müller-Riemenschneider

**Affiliations:** 1 Centre of Excellence for Soldier Performance, Singapore Armed Forces, 1 Selarang Ring Road, Block 2 #02-02, Singapore, 507087 Republic of Singapore; 2 Saw Swee Hock School of Public Health, National University of Singapore, 12 Science Drive 2, #10-01, Singapore, 117549 Republic of Singapore; 3 Yong Loo Lin School of Medicine, National University of Singapore, 10 Medical Drive, Singapore, 117597 Republic of Singapore; 4 Digital Health Center, Berlin Institute of Health, Anna-Louisa-Karsch-Straße 2, D-10178 Berlin, Germany

**Keywords:** Young men, Overweight & obesity, Weight loss maintenance, Motivations to exercise, BREQ-3

## Abstract

**Background:**

Physical activity is a critical component of lifestyle interventions to reduce body weight and maintain weight loss. The goal of this study was to examine the motivations to exercise in young men following a 5-month residential weight loss programme conducted in the Singapore military as part of National Service.

**Methods:**

We conducted a sequential mixed methods study starting with three focus groups comprising 21 programme instructors. Fifteen former programme participants aged 20.8 years (±1.4) with an average body mass index (BMI) of 29.3 kg/m^2^ (±4.6) were interviewed in-depth over a total duration of 9 h. Another 487 current programme participants aged 20.8 years (±1.1), BMI 27.1 kg/m^2^ (±2.6), completed a survey on weight loss, physical fitness, and motivations to exercise using the Behaviours Regulating Exercise Questionnaire (BREQ-3). Qualitative data was coded thematically using the six constructs of exercise motivation described by self-determination theory: amotivation, external, introjected, identified and integrated regulation and intrinsic motivation. Quotes from interviewees were cross-tabulated according to their weight maintenance trajectories. BREQ-3 responses were analysed according to initial body mass index (BMI), percentage weight loss and fitness.

**Results:**

Over the course of the residential programme interview and survey participants experienced an average weight loss of 15.6 kg (±6.5) and 13.0 kg (±5.4) respectively. Among the fifteen interviewees seven had gained no more than 34% of initial weight loss 6 months after completing the programme while another eight had gained more than 51%. We elicited three key themes from the data: (1) Barriers to exercise; (2) diminishing extrinsic motivation; and (3) unidentified exercise benefits. The integration of findings uncovered reinforcing motivational patterns in the areas of health, fitness, camaraderie and identified regulation. Narratives of self-acceptance and shift-work environments gave rise to potentially deleterious motivational patterns. Our findings suggest that successful transition from a residential programme to independent weight management requires a more deliberate pivot from predominantly extrinsic to intrinsic motivational approaches.

**Conclusion:**

Residential programmes such as the one investigated here, should develop a deliberate transition strategy, replace weight loss targets with physical performance goals and promote sports that are appropriate for young men affected by overweight and obesity.

**Supplementary Information:**

The online version contains supplementary material available at 10.1186/s12889-021-10373-z.

## Background

Physical inactivity and high body-mass index (BMI) are among the top five risk factors contributing to the global burden of disease [1, 2]. The two phenomena are also intimately linked [3]. Low levels of physical activity exacerbate positive energy balance and adiposity [4]. Conversely, a high body mass has been identified as a physical and psychological barrier to exercise participation [5, 6]. Evidence shows that the health of persons affected by overweight or obesity can be improved by increasing physical activity and/or losing weight [7, 8]. Health interventions targeting obese individuals, however, will typically feature exercise and dietary restrictions, or some combination of the two, to drive weight loss as the primary objective [9–11]. At the end of structured programmes, many experience difficulties keeping the weight off and face the disappointment of losing control [12–14]. In fact, to prevent relapse a person must spend 150 to 250 min per week exercising at moderate intensity [15]. This level is higher than the amount prescribed for substantial benefits in the health – at least 150 min per week - meaning that some health interventions might have set too stringent a goal of losing weight and keeping it off [16, 17].

It has been widely recognised that biomedical, social and behavioural factors drive the risk of relapse and weight gain [18–20]. While men are less likely than women to join a weight loss programme voluntarily, a man’s risk of drop-out is lower, especially when interventions are set in a context of sport and delivered in social settings [21]. Intensive programmes that target men therefore ought to emphasise the importance of regular physical activity and develop motivations that will have a lasting impact on exercise behaviour and long-term health.

Self-determination theory (SDT) provides a theoretical construct that allows us to characterise the diverse motivations to exercise as a spectrum of regulations from extrinsic to autonomous [22]. The key benefit of SDT is that it goes beyond the individual to examine the social and contextual factors that either promote or prevent the engagement in exercise thus identifying potential avenues for intervention [23]. Moreover, in the area of weight loss, it has been proposed that self-perceived autonomy, as framed by SDT, is a key predictor for long-term weight control [24]. SDT also forms the basis of the behavioural regulations in exercise (BREQ-3) questionnaire which characterises motivation to exercise based on six constructs: amotivation, external, introjected, identified and integrated regulation and intrinsic motivation (see Table 1) [25, 26]. The questionnaire and its earlier versions, BREQ and BREQ-2, have been validated in obese adolescents and youth to examine the relationship between motivation and exercise [27, 28].

**Table 1:** Behavioural Regulations of Exercise Questionnaire 3 (BREQ-3) Constructs

BREQ-3 Regulation Domain	Definition
Amotivation	A state of lacking the intention to act
External regulation	Exercise behaviour controlled by rewards and threats
Introjected regulation	Feelings of worth, for example pride, shame or guilt as a result of a particular behaviour
Identified regulation	Conscious valuing of a benefit of exercise
Integrated regulation	Exercise behaviour that was entirely assimilated as part of the genuine self
Intrinsic motivation	Inherent pleasure and satisfaction of participating in exercise behaviours

These concepts are put into practice on a daily basis in Singapore where all male citizens and permanent residents enter into 2 years of National Service upon completing post-secondary education. Personnel who meet the World Health Organisation’s BMI cut-off point for public health action in Asian populations (27.5 kg/m^2^) are enrolled in an intensive 5-month residential weight loss programme that is integrated into basic military training [29]. The programme is administered by a team of military leaders and administrators supported by civilian fitness trainers on an island location [30]. The goal of the programme is to reduce dietary intake, progressively increase levels of physical activity and induce a negative energy balance [31]. Physical fitness is tracked using the individual physical proficiency test (IPPT) comprising push-ups, sit-ups and a 2.4 km run [32]. The test is mandatory for all National Service personnel and it is scored over 100 points according to a predetermined matrix; a score of 50 points denotes that a participant has met the minimum fitness requirement.

A previous cross-sectional survey conducted in our residential programme showed that demographic factors, ethnicity, family history, physical activity and nutritional behaviours were associated with high initial body mass [33]. Mean weight loss in the programme was 12.5 kg [34]. Recent data have shown that most participants will experience at least 40% weight regain over the course of 1.5 years (Gorny AW: PES B(P) class graduating Dec 2016, unpublished). While the highly structured environment of our programme might seem unique, total duration and outcomes are similar to those seen in the most intensive lifestyle intervention programmes [35–37]. Features that set our study population apart from the literature are male gender, young age and mandatory enrolment. Still, any new insights into motivations to exercise in this key demographic could inform implementation strategies aimed at creating lasting public health impact.

The aim of this study was two-fold: To gather different perspectives on how the residential programme shaped the motivations to exercise; and to elicit key motivational patterns that prevented or enabled sustainable exercise habits.

## Methods

**Fig. 1:**
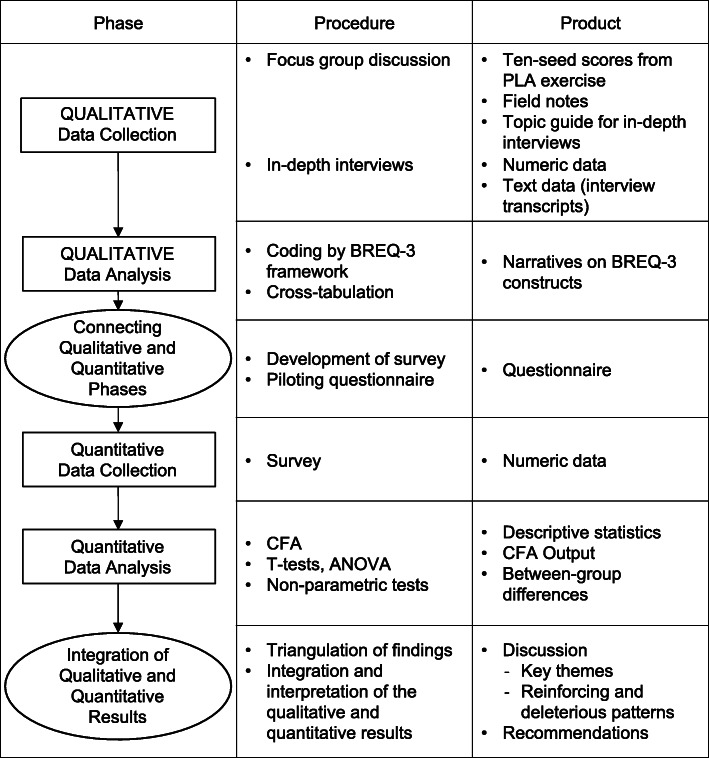
Visual Model Summarising Mixed Methods Data Procedures and Products. Legend: ANOVA: Analysis of variance. BREQ-3: Behavioural regulations in exercise. CFA: Confirmatory factor analysis. PLA: Participatory learning and action

This study utilised a sequential mixed methods design underpinned by a phenomenological theoretical approach. The study comprised qualitative exploration through focus groups and in-depth interviews followed by a quantitative cross-sectional survey. Figure 1 summarises the phases, procedures and products of data collection and analysis [38]. The study protocol was approved by the Defence Science Organisation - Singapore Armed Forces Institutional Review Board, Reference Number 0002/2019.

### Qualitative methods

The qualitative component of our study was conducted from March to August 2017 in military installations across Singapore. Our research team comprised one female Masters of Public Health (MPH) student and one male military physician pursuing a PhD. Both researchers had experience in qualitative methods, neither had a history of excess body weight. Analysis was guided and supported by two senior co-authors with expertise in qualitative methods and physical activity promotion.

To elicit the instructors’ perspectives we scheduled three separate focus groups involving a convenience sample of five to nine instructors each. Eligible discussants would have instructed in at least one full run of the residential programme. The focus groups were conducted onsite in a meeting room each lasting approximately on hour. We maintained the integrity of groups, meaning military leaders, fitness trainers and ancillary staff would not mix. The facilitator gave a short three-minute overview highlighting the problem of weight gain following the residential programme. Using a topic guide developed by our team (see Supplementary Table 1) the facilitator subsequently prompted participants to share their thoughts on what could be done differently to prevent relapse. A second member of the research team observed and took hand-written notes. The facilitator recorded key issues on a large sheet of poster paper for all participants to review. The session was concluded with a participatory learning and action exercise where participants were allowed to rank order and confirm key priorities in weight loss management using a ten-seed technique, which has been shown to stimulate participation and foster group consensus [39]. Audio-recordings were not made in order to promote more open sharing by participants who were all employees of the military.

To explore participant experiences further, we recruited a second convenience sample of 20 individuals who were randomly selected from the list of trainees who had completed the programme in December 2016. Our aim was to interview participants in-depth 6 months after completion of the programme within a 6-week period. Starting in May 2017 we wrote to each of their superior officers to schedule interviews that would be conducted behind closed doors near the place of work. Participation was voluntary and informed consent was required. No incentives were provided for participation in the interviews.

Key priorities identified by the focus groups informed the topic guide (see Supplementary Table 2) used in the in-depth interviews. The introduction to the study started with the process of obtaining informed consent. Subsequently the participant and the two researchers conducted a short ice-breaker comprising a brief self-introduction and an exercise of taking each other’s weight on a portable scale. Thereafter one researcher commenced the interview while the second team member observed and compiled field notes. All interviews were recorded on an audio device. The topic guide was structured to cover the interviewees’ lifestyles with an emphasis on physical activity and diet before, during, and after the residential programme. Participants were encouraged to reflect on their school, work and home environments. Before concluding the interview, participants were asked if they had any questions or concerns. After each interviewee was dismissed the researchers took time to reflect on the field notes and identify how the data added to the overall narrative. This process was refined over the first three interviews and ultimately helped determine when saturation had been reached.

Data from the focus groups and interviews were organised using the NVIVO 12 software package. The qualitative dataset was coded by one researcher using a thematic framework (see Table 1) comprising the six constructs of exercise regulations proposed by Deci & Ryan and embedded in the BREQ-3 instrument [40–42]. Interviewees were categorised, based on their weight trajectories as maintainers or relapsers, where a maintainer was defined as not having regained more than 50% of weight lost during the programme. The cross-tabulation function in the NVIVO 12 was used to compile relevant data for comparative analyses.

### Quantitative survey methods

We compiled a survey instrument that would match the key themes identified in the preliminary analyses of qualitative data. The initial questionnaire contained segments on demographic, lifestyle (for example smoking and eating habits), health data (height and weight), the 24-item BREQ-3 and the participants’ most recent fitness test results. The items on the BREQ-3 segment were rated on a Likert scale of five options ranging from ‘not true for me’ to ‘very true for me’. The questionnaire was designed to be anonymous and self-administered by a participant comfortable in the use of the English language. Height, weights and fitness test results would be self-reported. The questionnaire was pilot-tested and refined with the help of age-matched conscripts supporting our team.

A full cohort of approximately 600 members of the residential programme were targeted for recruitment during the penultimate week of the residential programme in February 2019. This period coincided with briefings by military staff in preparation for the dispersal of programme participants to various roles following basic training. Conscripts were invited by an independent member of staff, not related to the residential programme or research team, to participate in the self-administered pen-and-paper survey. Potential participants were informed that the survey was anonymous, that their responses would not be tracked and that there would be no direct incentives for their contribution. Persons who declined to participate were advised to return an empty questionnaire at the end of the session.

### Data analysis

BREQ-3 items were subjected to confirmatory factorial analysis (see Supplementary file 1) to test whether the six-factor structure adequately fit the data collected from our population as described in two recent publications [43, 44]. Then we computed composite scores deriving numerical averages for each item under the six BREQ-3 constructs. Summaries of survey items and the composite scores ranging from a minimum 1.0 to maximum 5.0 were compiled as parametric outcome data. Initial BMI was classified into overweight and obese categories using the WHO’s international cut-off value of 30 kg/m^2^. We categorised weight loss according to three groups signifying less than 10, 10 to 15% and more than 15% weight loss. The fitness test results were treated as a binary variable according to whether the participant had achieved 50 points or higher during the final test administered at the end of the programme. Our epidemiological analyses examined the relationship between BREQ-3 scores and binary exposure variables – BMI and IPPT results – using independent t-tests. The relationship between BREQ-3 scores and the three weight loss categories was examined using ANOVA. Where parametric statistical tests showed differences greater in significance than *p* < 0.05, analyses were repeated under the assumption of non-parametric outcome data using the Wilcoxon rank-sum and Kruskal-Wallis tests respectively. Our reporting of comparative analyses reflects the outcomes of non-parametric statistical tests exclusively. We used the STATA 14 programme for all statistical analyses.

We have used the Consolidated Criteria for Reporting Qualitative Research (COREQ) [45] and the Good Reporting of a Mixed Methods Study (GRAMMS) [46] checklists to ensure the completeness of this manuscript.

## Results

Our team recruited three focus-groups comprising six military leaders, eight fitness trainers and seven ancillary staff. Another sixteen graduates of the residential programme comprising ten transport operators, four security personnel and two junior officers provided informed consent to participate in in-depth interviews. Four potential interview candidates cited a lack of interest as a reason to decline participation. One participant had to be excluded from analysis as he had not completed the full residential programme following an injury. We collated fifteen audio recordings lasting between 25 and 55 min which were transcribed verbatim by the two researchers. For the quantitative survey we reached out to 589 current residential programme participants of whom 487 (82.7%) completed the questionnaire. Approximately 2% of data fields were returned incomplete or void and were coded as missing data.

Key characteristics of the overweight and obese men in our study population are summarised in Table 2. With the exception of two female fitness coaches involved in the focus groups all study participants were male and approximately 21 years of age. One third of participants reported a history of smoking and more than 85% of participants had attained post-secondary education. The average participant in the qualitative and quantitative samples had lost, 14.6 kg (15%) and 12.9 kg (14%) of body weight respectively. The average interviewee had gained 5.0 kg 7 months after completing the programme. Among the fifteen interviewees, eight regained 51 to 112% of weight lost during the programme and were classified as relapsers. The remaining seven gained no more than 34% of weight lost and were classified as maintainers.

**Table 2:** Characteristics of In-depth Interview and Survey Participants

		In-depth Interviews *N* = 15	Survey *N* = 487
Mean age in years (SD)		20.8 (1.4)	20.8 (1.1)
Highest Level of Education Attained (%)	Primary or Secondary	2 (13)	22 (4.5)
	Certificate of Technical Education	6 (40)	64 (13.1)
	Pre-University (‘A’ Levels)	3 (20)	6 (1.2)
	Polytechnic Diploma	4 (27)	395 (81.1)
Smoker (%)		5 (33.3)	169 (34.7)
Fitness Test Score ≥ 50 points (%)		9 (60)	248 (51)
Mean Height in m (SD)		1.75 (0.07)	1.73 (0.06)
Mean Body weight in kg (SD)	Initial	98.9 (15.3)	94.1 (12.6)
	Month 5	83.4 (11.6)	81.2 (9.9)
	Month 12	89.3 (14.0)	–
Mean BMI in kg/m^2^ (SD)	Initial	32.7 (4.3)	31.2 (4.2)
	Month 5	27.4 (3.8)	27.1 (2.6)
	Month 12	29.3 (4.6)	–

*SD* Standard deviation

*BMI* Body mass index

### Qualitative components

The priorities expressed by the three focus groups comprising fitness trainers (*n* = 8), military leaders (*n* = 6) and ancillary staff (*n* = 7) are summarised in Table 3 showing a predilection for extrinsic controls to drive exercise and dietary behaviours. Among ancillary staff, however, the need to cultivate better habits, hence autonomous regulation, was given highest priority. Specific quotes from in-depth interviews have been cross-tabulated (see Supplementary Table 5). Analysis of qualitative data revealed the following narratives on motivations to exercise.

**Table 3:** Key Priorities in Weight Maintenance Identified in Focus Groups Using Ten-Seed Method

Priorities to improve weight maintenance:	Number of Seeds Placed to Denote Priorities		
	Fitness Trainers *N* = 8	Military Leaders *N* = 6	Ancillary Staff *N* = 7
**Organisational (Extrinsic Regulation)**			
Incentives / Disincentives	3	2	–
Workplace Fitness Programmes	2	–	2
Parents / Families / Friends	2	2	–
Control Food / Drinks	–	3	1
Weight Monitoring	–	2	–
Peer Support	–	1	–
**Individual (Autonomous Regulation)**			
Cultivate Good Habits	–	–	4
Education and Resources	2	–	1
Motivations to Lead Healthy Lifestyles	1	–	1
Stress Management	–	–	1

#### Amotivation

Programme instructors described how sedentary lifestyles were common among recruits prior to their enrolment in the programme. Low physical fitness was identified as an important barrier to exercise. To overcome amotivation, the instructors agreed that a habit of exercising had to be cultivated in childhood and early adolescence. During the in-depth interviews, maintainers (*n* = 7) described amotivation as a consequence of fatigability, time pressure or chronic health issues such as asthma. They deduced that the residential programme had allowed them to overcome some of these barriers and had introduced them to the benefits of a physically active lifestyle. Relapsers (*n* = 8) described either high levels of inertia or intense discomfort when engaging in exercise. After completing the programme, these participants realised a decline in fitness levels resulting in renewed physical and psychological barriers when attempting to resume exercise routines.

#### External regulation

Instructors explained that geographical isolation, hierarchy of persons and strict discipline were key features of the 5-month residential programme. They felt that systems of external regulation such as weight monitoring, incentives and disincentives ought to be perpetuated beyond the residential programme to maintain the new lifestyle behaviours. Interviewees agreed that informal punishments in the form of remedial training were both a powerful deterrent and remedy for the minor infraction of weight regain. However, relapsers generally held more critical views of authority. One recalled a situation of profound embarrassment when a friend was singled out for punishment.

*[Witnessing his friend struggling] … “The trainers will be like, “If [at] any point in time I see this guy walk, every one of you will run extra. Five rounds!” (015 – relapser)*

For comparison, maintainers described fitness trainers as role models whose stern demeanour helped instil self-discipline. All interview participants were cognisant of the fact that they were at risk of weight regain because external regulation had ceased.

#### Introjected regulation

Instructors spoke of a sense of community fostered by the residential programme which was lost in the transition to new workplaces at the end of 5 months. Several interviewees recalled how for the first time they had developed a peer-group of overweight and obese males who could serve as a source of mutual encouragement. Relapsers shared that they lacked the motivation to exercise alone after the programme had ended. When asked whether this sense of camaraderie could be rekindled at a later stage, one maintainer disagreed. He felt that the sense of shame and regret brought about by weight rebound would only be amplified once it was evident that others were more successful at controlling their weight.

#### Identified regulation

Instructors assessed that more education outlining the risks associated with overweight and obesity was unlikely to achieve significant impact. Interviewees rarely mentioned personal health benefits of weight loss and active lifestyles and were more inclined to speak of family members who suffered from non-communicable diseases.

*“ … my entire family has issues with their weight. Just like me, they are all obese and do not seem to care about it. As such, I feel that I am currently in a precarious position. It is very unhealthy for all of us when we do not take care of our weight.” (002 – relapser)*.

Instead, interviewees described changes in physical appearance and the ability to wear normal-sized clothing as motivations to exercise. However, this sense of self-consciousness concerning physical appearance was so pronounced that one relapser felt unable to visit the gym. Another relapser reflected how his parents were more concerned that he was happy, regardless of his external appearance. For comparison, maintainers tended to identify improved fitness as a source of motivation which in turn enabled for increased participation in sports and exercise.

*“Usually, my stamina is not very good. Every time I participate in activities of reasonably high intensity, I have to take unnecessarily prolonged rests. My goal is to become fitter so that I can participate more in activities which interest me.” (010, maintainer)*

#### Integrated regulation

Instructors agreed that while exercise was an integral part of military life, proactive measures were needed to sustain weight loss after leaving the programme. Interviewees explained that jobs in transport and logistics were sedentary in nature while other military vocations, such as the infantry, were more physically engaging. Road safety regulations in fact required drivers to observe periods of mandatory rest between duties. For comparison, two maintainers shared that a high level of physical fitness was wedded to their identities as military leaders.

*“I have to keep fit, otherwise my [subordinates] will look down on me.” (014, maintainer)*

#### Intrinsic regulation

Instructors shared that admission into the residential programme was a clear indicator of low intrinsic motivation to exercise. They stressed that even after the residential phase exercise had to be cultivated through extrinsic regulation. Five interviewees recalled their keen involvement in sports during childhood. In adolescence, however, the pressures of school and part-time employment took on a higher priority than active lifestyles. Only two participants, both of whom were maintainers, reflected on any form of enjoyment born out of participating in sports and exercise.

### Quantitative component

Confirmatory factorial analysis results (see Supplementary file 1) supported adequate convergent and discriminatory validity of the BREQ-3 scales in our sample. Overall, survey respondents rated items describing amotivation lowest of all six constructs (Table 4), followed by external then integrated regulation. Items describing identified regulation were scored highest with intrinsic motivation and introjected regulation ranked second and third respectively. This rank order did not differ when data were stratified by initial BMI or weight loss categories. However, the rating of all six constructs differed significantly when stratified by fitness test result; fitter respondents expressed stronger agreement with constructs of autonomous regulation (introjected, identified, integrated and intrinsic) and rejected statements of amotivation and external regulation.

**Table 4:** Motivations to Exercise – Mean Composite Scores with Standard Deviations at End of Residential Programme, Overall, by Initial BMI category, Percentage Weight Loss and Fitness Test Results

	Overall	Initial BMI Category		Weight Loss			Fitness Test Result	
	–	< 30 kg/m^2^	≥30 kg/m^2^	< 10% loss	10–15% loss	> 15% loss	<50pts	≥50pts
N (%)	487 (100)	183 (38)	304 (62)	99 (20)	203 (42)	185 (38)	239 (49)	248 (51)
Amotivation (SD)	1.7 (0.7)	1.7 (0.8)	1.7 (0.7)	1.7 (0.8)	1.7 (0.7)	1.6 (0.6)	1.8 (0.8)	1.6 (0.6)^$^
External Regulation (SD)	2.3 (0.8)	2.3 (0.8)	2.3 (0.8)	2.2 (0.8)	2.2 (0.8)	2.4 (0.9)	2.4 (0.9)	2.2 (0.8)
Introjected Regulation (SD)	3.0 (1.0)	3.1 (1.0)	3.0 (1.0)	3.0 (1.0)	3.0 (1.0)	3.1 (1.0)	2.8 (1.0)	3.2 (0.9)*
Identified Regulation (SD)	3.7 (0.7)	3.7 (0.7)	3.7 (0.7)	3.7 (0.8)	3.7 (0.7)	3.7 (0.6)	3.5 (0.7)	3.9 (0.7)*
Integrated Regulation (SD)	2.9 (0.9)	2.9 (1.0)	2.9 (1.0)	2.8 (1.0)	2.8 (1.0)	3.0 (0.9)	2.6 (0.9)	3.2 (0.9)*
Intrinsic Motivation (SD)	3.3 (0.9)	3.3 (0.9)	3.3 (0.9)	3.3 (0.9)	3.3 (0.9)	3.3 (0.8)	3.0 (0.8)	3.6 (0.8)*

*BMI* Body mass index

*BREQ-3* Behavioural Regulations in Exercise Questionnaire 3

^$^ *p* < 0.01

* *p* < 0.001

## Discussion

### Motivations to exercise

While our study design was exploratory and sequential in nature, our analyses were explanatory and convergent [47]. Using triangulation within qualitative and quantitative data sources [48] we elicited three key themes on the motivations to exercise: (1) persistent barriers to exercise; (2) diminishing extrinsic motivation; and (3) unidentified benefits of exercise.

### Persistent barriers to exercise

While the residential programme had suppressed exercise amotivation, it was unable to eliminate physical and social barriers to exercise that resurfaced after transition. The literature on exercise prescription advocates a structured approach to raising levels of physical activity through step-wise increases in exercise duration, then frequency and lastly intensity [49]. The pain and discomfort experienced by relapsers in our study might be interpreted as an inability to tolerate a large sustained increase in the level of activity resulting in over-training and increased risk of illness and injury despite best efforts to ensure gradual progression [50]. Concerning social barriers to exercise, environments that stigmatise persons suffering obesity can foster a strong sense of exercise avoidance [51]. In our sample, introjected regulation through camaraderie generally helped participants overcome the fear of engaging in exercise. However, as is the case with conventional weight loss programmes, this sense of community either receded or was completely lost after transition [52].

Consistent with the literature our survey findings suggest that the relationship between fitness and motivations to exercise was likely a direct one not mediated by initial BMI or amount of weight lost [53, 54]. While we are unable to comment on the direction of the relationship, this finding suggests that interventions to promote autonomous motivations to exercise might be better directed at the least fit and not the heaviest segment of the population. Interventions which have treated activity or fitness as the primary outcome and relegated weight loss to a secondary outcome have gained traction in recent years [55]. For men such approaches have the advantage of leaning on common perceptions of masculinity while weight loss maintenance and slimming typically evokes femininity [56].

### Diminishing extrinsic motivation

The success of residential programmes has been attributed to the structured environments they can build around the needs of persons suffering overweight and obesity [57, 58]. Our data even suggests that there was a demand for a framework of incentives and penalties that extended past the end of the programme. Previous systematic reviews have shown that the impact of financial incentives for weight control in obesity was at best unclear [59, 60]. A rigorous trial in military veterans showed that financial incentives could produce tangible results, nevertheless the risk of relapse was high after disbursement had completed [61]. One novel approach to promoting exercise under free-living conditions involves mobile health interventions [62]. A recent randomised controlled trial showed that activity trackers administered in a workplace context on their own helped sustain a higher level of physical activity, even in absence of overt incentives [63].

### Unidentified benefits of exercise

Our survey data indicated that programme participants agreed most strongly with identified regulation as a motivation for exercise, meaning that they would have assigned a conscious value or benefit to exercise. From the qualitative data we were able to elicit two sub-themes detailing this benefit: (1) to control body weight and (2) to improve physical fitness. The former carried the outward benefit of a leaner, healthier appearance. The latter, however, was recognised almost in hindsight, as an enabler of exercise participation. A minority of voices discussed chronic disease risks but tended to recognise these as late consequences of high body weight, not inactive lifestyles.

Current scientific consensus is that long-term weight loss requires a combination of diet and exercise [64]. Exercise alone has been shown to produce smaller, more short-lived changes in weight [65]. The strong focus on body weight in our sample suggests an incomplete appreciation for the direct benefits of exercise that include improved chronic disease risk and better mental health [66, 67].

### Integration of quantitative and qualitative data

Our qualitative findings seem to suggest that while the programme helped participants overcome barriers to exercise, it did not prepare them for independent weight management. To do so would require a deliberate pivot from predominantly extrinsic to a more autonomous motivations to exercise, where the benefits of exercise would help sustain an active lifestyle. More importantly, our quantitative findings did not fully converge onto this picture as we uncovered that the young men in our population might have been at risk of overestimating their propensity for autonomous regulation. Therefore, to help explain this discrepancy, we integrated quantitative with qualitative results to discuss key reinforcing and deleterious patterns across different BREQ-3 constructs [68].

### Reinforcing patterns

Low fitness and undesirable physical appearance were recurring themes in our study. High body weight before the programme and relapse after transition would produce either discouragement, physical discomfort or social distress, which in turn would reinforce inactive behaviour. Conversely, improved fitness and lean appearance were constructed as pre-requisites to enhanced enjoyment of exercise and sports, thus reinforcing the motivation to exercise. It has been suggested that in order to promote greater exercise autonomy, exercise programmes should avoid body weight and physical appearance as motive [69]. A framing that focuses on active lifestyles and fitness might foster stronger identified and introjected motivations to exercise. This could be achieved for example by monitoring of strength and cardiorespiratory fitness or by using gadgets and smartphone technologies to track activity levels [70, 71]. Finally, medical literature tends to favour health risks as vehicles for identified regulation [72]. Our study participants were more likely to register the real problems of disease in family members rather than in their distant-future selves. A significant minority of participants even shared their experiences with premature death due to noncommunicable disease in the family. These first-hand experiences could influence a personal sense of vulnerability and offer themselves as relevant topics for health promotion messaging [73].

### Deleterious patterns

From the qualitative data it emerged that young men living with obesity had to negotiate a fine balance between asserting self-esteem and responding to external cues that encouraged weight loss. A strong sense of camaraderie in the programme helped alleviate the social stigma of obesity. However, it might have inadvertently prevented a critical discussion on the utility and risks of negative self-image in overweight and obese individuals [74–76]. Prevalent framing of changes in appearance as a source of inspiration and identified regulation in this population offers an opportunity to deepen the discussion on how to promote healthy lifestyles without diminishing self-esteem. One alternative avenue could be to emphasise the tangible benefits of exercise over the intangible improvements associated with lower body weight [77].

In our qualitative sample weight gain trajectories were related to the nature of work participants engaged in after completing the residential programme. Transport drivers routinely prioritised work obligations over their own needs leading to a permanent disruption of meal and exercise routines. These findings were in keeping with patterns observed in UK adolescents transitioning into adulthood [78]. Considering that a significant number of former participants were assigned to roles in security and logistics, it follows that success in transition might be bolstered by interventions at specific workplaces. Finally, it should not be overlooked that interviewees generally and begrudgingly accepted exercise as a legitimate means of instilling discipline. By associating exercise with punishment, however, the programme might have inadvertently suppressed the development of autonomous motivations to exercise, as reflected by participants who lamented the loss of strong external regulation after transition.

### Strengths

A key strength of this study was its mixed methods design, which allowed us to examine diverse lived experiences and assess the magnitude and frequency of motivations to exercise [79]. Our findings in a military population might lack generalisability to community-based weight loss programmes, the study nevertheless offers an opportunity to better understand the after-effects of an intensive lifestyle-intervention programme. Furthermore, our programme was designed to meet the needs of a wide segment of the population, including participants who might not have been captured at all by a voluntary programme.

### Limitations

Our study was designed to explore a specific period of transition with a focus on exercise. This meant that our methods prevented us from probing deeper into sensitive psychosocial themes such as bullying which constitutes a significant part of the literature on youth suffering from obesity [80]. Furthermore, we used a deductive approach framed by self-determination theory, thus limiting our ability to explore alternative behavioural constructs. The selection of participants for the qualitative study was through convenience sampling, which might not have provided the same depth and clarity as key-informant and snow-ball selection techniques. There were non-responders in both the qualitative and quantitative portions of the study who might concealed important data that would have expanded the proposed narrative. Finally, it was not feasible to review our results with the study participants meaning the participants themselves were not able to lend support to, or refute our findings and recommendations.

## Conclusions

We have established that short-term benefits produced by strong external regulation had to be reconciled with the long-term risks the residential programme inflicted on autonomous motivations to exercise. More specifically, we have elicited reinforcing and deleterious motivational patterns that could inform future efforts to reform the residential programme. Overall, our study suggests that the transition from a 5-month residential programme to successful independent weight management might require a deliberate pivot from predominantly extrinsic to intrinsic motivational approaches.

### Recommendations

The findings of data triangulation and integration have allowed us to formulate the following recommendations which would help the residential programme reframe its approach to exercise and physical activity promotion:

1. To develop a strategy which allows participants to deliberately transition from external regulation to more autonomous exercise activities over the course of the 5-month programme. Such an approach would retain the initial benefits of powerful external regulation and supportive communal settings. It would educate participants on the dangers of an inactive lifestyle and progressively cultivate sport as an effective means of sustaining higher levels of activity after transition.
2. To replace weight loss targets with physical performance goals. The main goal of the programme could be to increase the levels of activity and achieve concomitant improvements in objectively assessed fitness. Weight maintenance, not weight loss, would then be identified as a secondary goal. Activity levels could be monitored longitudinally through by means of electronic tracking while fitness testing could be retained.
3. To promote lower-impact sports which are appropriate for youth with higher body weight, for example cycling, rowing, weight-lifting or martial arts. These sports could be introduced during the programme and participation could be promoted at workplaces. This approach would allow participants to reduce the risk of injury and in certain sports even experience the competitive advantage of a large body shape.

## Supplementary Information

**Additional file 1: Supplementary Table 1.** Topic guide for focus group discussion

**Additional file 2: Supplementary Table 2.** Topic guide for in-depth interviews

**Additional file 3: Supplementary File 1.** Confirmatory Factorial Analysis. Methods, results and references for confirmatory factorial analysis of BREQ-3 constructs with Supplementary Tables 3 and 4 and Supplementary Figure 1

**Additional file 4: Supplementary Table 5.** In-depth interview quotes relating to exercise behaviours cross-tabulated by weight loss trajectory

## Data Availability

The quantitative datasets used and/or analysed during the current study are available from the corresponding author on reasonable request. The qualitative transcripts and datasets generated and/or analysed during the current study are not publicly available due to participant confidentiality but are available from the corresponding author on reasonable request.

## Abbreviations

ANOVA: Analysis of variance
BMI: Body mass index
BREQ-3: Behavioural regulations in exercise questionnaire - 3
CFA: Confirmatory factor analysis
COREQ: Consolidated criteria for reporting qualitative research
GRAMMS: Good reporting of a mixed methods study
IPPT: Individual physical proficiency test
MPH: Masters of public health
SD: Standard deviation
SDT: Self-determination theory
