# Supplementary material for: Motivations to exercise in young men following a residential weight loss programme conducted in National Service - a mixed methods study
Source: BMC Public Health. 2021 Feb 17;21:370. doi: 10.1186/s12889-021-10373-z (PMC7890904; doi:10.1186/s12889-021-10373-z)
Supplement: Supplementary file 1 — Additional file 1: Supplementary Table 1. Topic guide for focus group discussion [file 12889_2021_10373_MOESM1_ESM.docx]

| Supplementary Table 1. Topic Guide for Focus Group Discussion | |
| --- | --- |
| Objective: | To unpack the residential programme graduates’ experiences on how/why weight loss is achieved/not achieved, and what could undermine sustainability of weight loss after the residential programme. |
| Instruction: | Use a chronological approach (past, present and future) |
| Critique: | Is the programme effective?  Do you have examples?  Barriers to weight loss maintenance?  Enablers of weight loss maintenance? |
| Understanding Impact | How do participants feel after losing weight?  How would they feel after relapsing? |
| Proposals | How should we improve the programme? |
| Closing | Any new ideas / insights / thoughts at the end of the session? |
