# Supplementary material for: Motivations to exercise in young men following a residential weight loss programme conducted in National Service - a mixed methods study
Source: BMC Public Health. 2021 Feb 17;21:370. doi: 10.1186/s12889-021-10373-z (PMC7890904; doi:10.1186/s12889-021-10373-z)
Supplement: Supplementary file 2 — Additional file 2: Supplementary Table 2. Topic guide for in-depth interviews [file 12889_2021_10373_MOESM2_ESM.docx]

| Supplementary Table 2. Topic Guide for In-depth Interviews | | |
| --- | --- | --- |
| Introduction | *Getting to know the interviewee*  Tell me about yourself.  What do you want to do when you finish national service? | |
| Before the Programme | What did you do before entering national service? | What were your meals like?  Did you … - usually exercise on your own?  - you play a sport?  - you think exercising was a habit? |
| During the Programme | Tell me about your time in the residential programme? | Do you think the how has the residential programme changed your …  - eating habits?  - exercise habits? |
| After the Programme | Tell me about you’re your current workplace (in National Service). | What are your meals like?  Did you … - usually exercise on your own?  - you play a sport?  - you think exercising is a habit? |
| Weight | Can you tell me how do you feel before and after losing weight? | How often do you monitor your weight now?  Why is your body weight important? |
| Social | Tell me about your friends.  What’s it like at home? | |
| Well-Being | How do you cope with hardship or stressful times?  What keeps you going? | |
| General | Can you tell me about your experience of…  You mentioned […] can we talk more about that?  What was your view on that?  How did you feel? | |
| Going Deeper | Could you give me an example?  Can you be more specific?  In what way?  Why do you say that?  Can you tell me more about that? | |
