# Supplementary material for: Motivations to exercise in young men following a residential weight loss programme conducted in National Service - a mixed methods study
Source: BMC Public Health. 2021 Feb 17;21:370. doi: 10.1186/s12889-021-10373-z (PMC7890904; doi:10.1186/s12889-021-10373-z)
Supplement: Supplementary file 3 — Additional file 3: Supplementary File 1. Confirmatory Factorial Analysis. Methods, results and references for confirmatory factorial analysis of BREQ-3 constructs with Supplementary Tables 3 and 4 and Supplementary Figure 1 [file 12889_2021_10373_MOESM3_ESM.docx]

**Supplementary File 1. Confirmatory Factor Analysis**

**Methods**

BREQ-3 items were subjected to confirmatory factor analysis (CFA) to test whether the six-factor structure adequately fit the data collected from our population as described in two recent publications.[1,2] Missing data in our sample were small (0.3%) so we chose not to omit or replace these scores. For the purposes of CFA and other subsequent analyses we assumed BREQ-3 scores were continuous variables that followed a normal distribution. We assessed goodness of fit using the comparative fit index (CFI), Tucker-Lewis index (TLI), and root mean squared error of approximation (RMSEA). We allowed CFI and TLI values >0.95 to indicate an acceptable fit [3] while RMSEA values less than or equal to 0.08 were considered satisfactory.[4] We screened for reliability using Cronbach’s alpha and Composite Reliability (CR) designating scores above 0.70 as acceptable.[5] We recognised internal consistency when the Average Variance Extracted (AVE) was 0.50 or greater.[6] We screened for discriminant validity by examining bivariate correlations with values of *r* greater than 0.85.[7] Discriminant validity was confirmed using the Fornell and Larcker criterion that proscribes that the square-root of a construct’s AVE should be greater than the value of its coefficients of bivariate correlation with the other constructs.

**Results**

Summary statistics on individual BREQ-3 items (Supplementary Table 6) demonstrated acceptable normality of score distribution for five of the six constructs with the exception of amotivation scores which were strongly skewed.

| Supplementary Table 3. Descriptive statistics for the behavioural regulation in exercise questionnaire (BREQ-3) items. | | | | | |
| --- | --- | --- | --- | --- | --- |
| No | Items | Mean | Standard  Deviation | Skewness | Kurtosis |
|  | Amotivation |  |  |  |  |
| 2 | *I don’t see why I should have to exercise.* | 1.75 | 0.96 | 1.28 | 4.22 |
| 8 | *I can’t see why I should bother exercising.* | 1.84 | 0.92 | 1.05 | 3.92 |
| 14 | *I don’t see the point in exercising.* | 1.52 | 0.77 | 1.56 | 5.49 |
| 20 | *I think exercising is a waste of time.* | 1.63 | 0.85 | 1.37 | 4.58 |
|  | External Regulation |  |  |  |  |
| 6 | *I exercise because other people say I should.* | 2.57 | 1.13 | 0.28 | 2.32 |
| 12 | *I take part in exercise because my friends / family / partner say I should.* | 2.73 | 1.15 | 0.10 | 2.29 |
| 18 | *I exercise because others will not be pleased with me when I don’t.* | 1.79 | 0.96 | 1.19 | 3.92 |
| 24 | *I feel under pressure from my friends / family to exercise.* | 2.05 | 1.08 | 0.75 | 2.64 |
|  | Introjected Regulation |  |  |  |  |
| 4 | *I feel guilty when I don’t exercise.* | 3.28 | 1.14 | -0.24 | 2.55 |
| 10 | *I feel ashamed when I miss an exercise session.* | 2.77 | 1.17 | 0.112 | 2.26 |
| 16 | *I feel like a failure when I haven’t exercised in a while.* | 2.97 | 1.26 | -0.05 | 2.03 |
| 22 | *I would feel bad about myself if I was not making the time to exercise.* | 3.10 | 1.17 | -0.17 | 2.25 |
|  | Identified Regulation |  |  |  |  |
| 1 | *It’s important to me to exercise regularly.* | 3.91 | 0.94 | -0.61 | 3.08 |
| 7 | *I value the benefits of exercise.* | 4.12 | 0.85 | -0.75 | 3.21 |
| 13 | *I think it is important to make the effort to exercise regularly.* | 4.09 | 0.87 | -0.86 | 3.74 |
| 19 | *I get restless if I don’t exercise regularly.* | 2.66 | 1.19 | 0.18 | 2.16 |
|  | Integrated Regulation |  |  |  |  |
| 5 | *I exercise because it is consistent with my life goals.* | 3.21 | 1.08 | -0.23 | 2.45 |
| 11 | *I consider exercise part of my identity.* | 2.60 | 1.12 | 0.32 | 2.45 |
| 17 | *I consider exercise a fundamental part of who I am.* | 2.74 | 1.12 | 0.14 | 2.31 |
| 23 | *I consider exercise consistent with my values.* | 3.03 | 1.07 | -0.14 | 2.50 |
|  | Intrinsic Motivation |  |  |  |  |
| 3 | *I exercise because it’s fun.* | 3.14 | 1.17 | -0.28 | 2.46 |
| 9 | *I enjoy my exercise sessions.* | 3.49 | 0.97 | -0.33 | 3.01 |
| 15 | *I find exercise a pleasurable activity.* | 3.15 | 1.03 | -0.13 | 2.69 |
| 21 | *I get pleasure and satisfaction from participating in exercise.* | 3.38 | 1.01 | -0.28 | 2.73 |

Goodness of fit statistics (χ^2^ (237) = 707.72, *p* < 0.001; CFI = 0.922; TLI = 0.909; RMSEA = 0.064) to the factorial structure confirmed the validity of the six-factor model in our sample. Factor loadings of the model ranged from 0.57 to 0.88 and loaded significantly in their respective constructs (Supplementary Figure 1) supporting measurement convergent validity.

| Supplementary Figure 1. Standardized parameters (factorial weights, covariance factors, and measurement errors) for the BREQ-3. |
| --- |
| 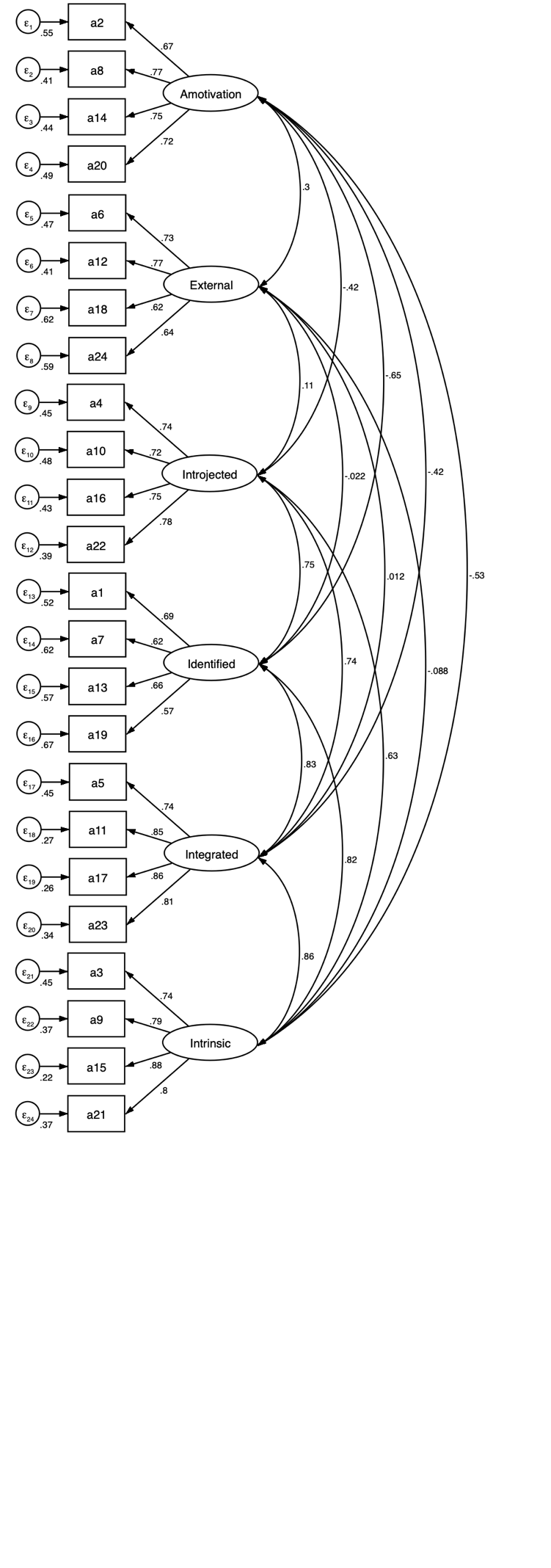 |
| Behavioural Regulations in Exercise Questionnaire 3 (BREQ-3)  All factor loadings were significant (*p* <0.05). |

The alpha and composite reliabilities (Supplementary Table 7) ranged from 0.64 to 0.89 and from 0.74 to 0.89 respectively which were considered adequate.

| Supplementary Table 4. Reliability, means, standard deviations and bivariate correlations in the behavioural regulations in exercise questionnaire (BREQ-3) subscales. | | | | | | | | | | | |
| --- | --- | --- | --- | --- | --- | --- | --- | --- | --- | --- | --- |
|  | μ | sd | α | CR | AVE | AM | EX | IJ | ID | IG | IM |
| Amotivation (AM) | 1.68 | 0.70 | 0.82 | 0.82 | 0.53 | 1.00 | - | - | - | - | - |
| External (EX) | 2.28 | 0.84 | 0.78 | 0.78 | 0.48 | 0.26^ | 1.00 | - | - | - | - |
| Introjected (IJ) | 3.04 | 0.98 | 0.84 | 0.84 | 0.56 | -0.34^ | 0.07 | 1.00 | - | - | - |
| Identified (ID) | 3.69 | 0.71 | 0.72 | 0.73 | 0.40 | -0.48^ | -0.02 | 0.61^ | 1.00 | - | - |
| Integrated (IG) | 2.89 | 0.95 | 0.89 | 0.89 | 0.67 | -0.37^ | 0.00 | 0.64^ | 0.70^ | 1.00 | - |
| Intrinsic (IM) | 3.29 | 0.89 | 0.64 | 0.88 | 0.65 | -0.45^ | -0.09 | 0.55^ | 0.68^ | 0.77^ | 1.00 |
| μ, mean; sd, standard deviation; α, Cronbach’s alpha; AVE, average variance extracted; CR, composite reliability. | | | | | | | | | | | |

In general, the AVEs of this study were above the recommended threshold of 0.50, with the exception of the identified regulation construct, which exhibited a value of 0.40. Bivariate correlations were below 0.85 in value indicating adequate discriminant validity which was confirmed using Fornell and Larkner criterion. The behavioural constructs located on the more autonomous spectrum of regulation showed moderately strong bivariate correlation. There was only weak correlation between amotivation and external regulation on the opposite end. These results supported adequate convergent and discriminatory validity of the BREQ-3 scales in our sample.

**References**

1. Zamarripa, J., Castillo, I., Baños, R., Delgado, M., & Álvarez, O. (2018). Motivational regulations across the stages of change for exercise in the general population of Monterrey (Mexico). Frontiers in psychology, 9.
2. Cid, L., Monteiro, D., Teixeira, D., Alves, S., Moutão, J., Teques, P., ... & Palmeira, A. (2018). The behavioural regulation in exercise questionnaire (BREQ-3) Portuguese-version: evidence of reliability, validity and invariance across gender. Frontiers in psychology, 9, 1940.
3. Hu, L. T., & Bentler, P. M. (1999). Cutoff criteria for fit indexes in covariance structure analysis: Conventional criteria versus new alternatives. *Structural equation modeling: a multidisciplinary journal*, *6*(1), 1-55.
4. Cole, D. A., & Maxwell, S. E. (1985). Multitrait-multimethod comparisons across populations: A confirmatory factor analytic approach. *Multivariate Behavioral Research*, *20*(4), 389-417.
5. Cronbach, L. J. (1951). Coefficient alpha and the internal structure of tests. *psychometrika*, *16*(3), 297-334.
6. Fornell, C., & Larcker, D. F. (1981). Structural equation models with unobservable variables and measurement error: Algebra and statistics.
7. Kline, R. B. (2015). *Principles and practice of structural equation modeling*. Guilford publications.
