# Supplementary material for: Motivations to exercise in young men following a residential weight loss programme conducted in National Service - a mixed methods study
Source: BMC Public Health. 2021 Feb 17;21:370. doi: 10.1186/s12889-021-10373-z (PMC7890904; doi:10.1186/s12889-021-10373-z)
Supplement: Supplementary file 4 — Additional file 4: Supplementary Table 5. In-depth interview quotes relating to exercise behaviours cross-tabulated by weight loss trajectory [file 12889_2021_10373_MOESM4_ESM.docx]

| Supplementary Table 5. In-depth Interview Quotes Relating to Exercise Behaviours Cross-tabulated by Weight Loss Trajectory. | | |
| --- | --- | --- |
| BREQ-3 Regulation Construct | Relapsers | Maintainers |
| Amotivation | *I am free every weekend. However, I am too lazy to leave my house. I tend to spend my free time at home instead of exercising. (002)*  *I want to exercise. In fact, I regularly plan to exercise. However, I also have a habit of procrastinating. (laughs). (008)*  *Before I joined National Service, I never exercised. At school, I never played any soccer. I tended to mainly play computer games. (008)*  *Because I injured [knee-cap tilt] myself, I cannot really run. (011)*  *I don’t like the discomfort of exercising. However, it is something you have to go through. There is a saying, “No pain no gain”. There are days when I am so tired from exercising that I am fatigued and do not want to move. (012)*  *So if you start all over again for PT (physical training), you will feel demoralised. You can’t be the same as you were before BMT (Basic Military Training). Your motivation will drop and you will be slower than the rest. (013)*  *Every week we do the same thing over and over again and it’s getting boring. (013)*  *Running works. But I’m not a running type of guy. (013)*  *When I first started, I suffered a lot…I actually have childhood asthma which caused significant difficulties in breathing and wheezing. (015)*  *I initially wanted to lose weight. However, choices and levels of motivation can change by the day. There are days when I want to lose weight, and there are days when I just feel too tired and lose motivation. (015)* | *It has always been ‘mind over body’. If you can convince yourself to go through all these exercises, you can achieve more than what you initially thought. (003)*  *If your force [overweight subordinates], they won’t perform. They aren’t motivated, they will not entertain you. They will not perform optimally. They will procrastinate. (007)*  *Sometimes you feel very lazy, right? You just don’t want to exercise. (010)*  *[Life before National Service] Once I wake up, I play computer games, eat, play computer games, eat and sleep. This cycle used to repeat every day. (007)*  *I had the mentality that I needed to do something about it. However, I was just too lazy. (007)*  *Before NS, I was really obese. I didn’t exercise much because I had*  *asthma since young. (010)*  *Before I enlisted I didn’t really enjoy exercise. It just felt really tiring. But after BMT, once I completed exercising, I would feel a sense of pleasure. (010)*  *A lot of people are lazy. They tend to choose the easier option when a choice is given to them. (010)* |
| External Regulation | *Whether I like it or not, I’m forced to do the physical exercises with my brothers around me. (004)*  *We have a rule. Every time we return to camp, after the weekend, if we gain half a kilo, we have to do push ups and sit ups. So everyone is really watching their weight and making sure they don’t gain as much and get these punishments. This rule drives fear into us. (004)*  *In your whole life you won’t push yourself to train so much. In army, you can push yourself, push your limit ... For me I don’t like running. I’ve been in the army for a year already and I still actively join [relay running race] training. This is despite not really liking to run. (006)*  *[After National Service] You won’t be running a lot due to other commitments. If you do not keep on running, you will definitely gain weight.*  *For example, I do push-ups. My push- ups [are] not so perfect. Even when we do 6-pointers (push-ups on hands and knees), we get scolded. They force us to do 4-pointers (conventional push-ups). (015)*  *[Witnessing a friend cry] The trainers will be like, “If any point in time I see this guy walk, everybody of you will run extra 5 rounds. (015)*  *They [instructors] scolded us because we never ran in pace with them. Yes, I never ran with them, but I was not selfish. My breathing was not so good. So I was jogging slowly. (015)* | *I don’t like the fitness trainers … because they like to give us extra exercise. But it’s good for us actually. (001)*  *Trainers … are very motivating. They always try to encourage us. Try to lose more weight, always think of creative exercises to play with. (007)*  *I used to be in the TAF (trim and fit) club during secondary school but I was still active in sports. (009)*  *I was [expletive] bored. Instead of picking up [the medicine ball], I flipped the ball using my leg. One of the sergeants saw, he told me to pick up and put down the medicine ball 100 times [drill]. (009)*  *I think I was pretty lucky. My [instructors] were very nice. They were also very motivational. A few of my [instructors] were previously obese also. They told us their story and we exercised together. (010)*  *I had this instructor - his trainings were the harshest, but they were the most enjoyable ones. … The way he puts us through the training, it was not torturous, but the process was enjoyable because of the way he trained us and the things he said. Sometimes he threw insults at us, but it was actually funny. (010)*  *I think that’s a pretty big reason why a lot of people gained weight when they moved to their own units. Because they don’t treat them like [weight loss programme participants]. … like when you do PT (physical training), if you fall behind, then they won’t really push you. They will just see that you’ve fallen behind. You have to push yourself. For some people that are not as mentally fortified to push themselves, they might just fall behind [participant shrugged]. (014)*  *Yeah, it’s kind of forced upon us. There is not much of a choice. But … I signed up for the 21km [race], so we have to clock a certain mileage. (014)* |
| Introjected Regulation | *Running … because you are the last one already, you confirm must run. (006)*  *Sometimes I go to the gym, following my friends there. (008)*  *You find a lot of the people who think the same as you. Everyone gathers together and work for it. Quite a few of us like really want to lose weight and we just train for it. (011)*  *My mum and my dad couldn’t bowl because they had [health] problems. So it was not fun, because only the children were playing and the parents not joining. We also used to play with kites at Marina Bay before they built the barrage there. After building the barrage, they don’t have the field for playing kites anymore. (012)*  *I had a friend who I used to run together with. It’s about their mind-set, if you want to lose weight or not. We don’t force them, we just advise them or encourage them. If we force them, they won’t like it and we will get into [a] fight. They will [definitely] ask me, “If u want to lose weight, you lose weight on your own. Don’t lose weight with me.” … It seems negative, but it’s not negative. (015)* | *I always tell myself I can do this so I just pushed myself all the way. Without knowing, I didn’t even realise my own change. (003).*  *Determined? I’m not so determined. I just go with the flow. (003).*  *When the whole company trains together, it’s fun. When a lot of your friends participate together, it is quite fun, quite motivating, quite challenging. We look forward to it. (007)*  *I bond quite well with them [friends]. We do things together. Every time we exercise, we will gather into one corner and will really train hard … that’s what makes it fun. (007)*  *I already paid for the gym membership, I should not waste money, I should go. That’s what made me go. Should not waste the money, just go. (007)*  *Actually, I learned [to gym] from my friends because I used to exercise with my friends. … During the [residential programme] period, they also motivated me. (010)*  *I think the company is very important, like the exercising company. You won’t really feel very motivated when you do it alone, but if you do it in a group, you won’t give up that easily. (010).*  *I would just follow my [instructor’s] pace and keep jogging with him. There was this one time we jogged for 45 minutes to an hour and I just kept following him. I just didn’t want to give up quite yet. For me, nothing ever seemed too hard to be able to do. It all seemed hard but doable. But mostly the hardness came from just being unfamiliar, because when you are on the ground doing it, you are just doing it. (014)* |
| Identified Regulation | *…my entire family has issues with their weight. Just like me, they are all obese and do not seem to care about it. As such, I feel that I am currently in a precarious position. It is very unhealthy for all of us when we do not take care of our weight. (002)*  *I gained weight when I did more driving and less exercising. And my shirts actually didn’t fit and I had to switch back to the bigger sizes. So you know, to wear nicer clothing, to look good, it motivates me a bit more. (004)*  *On the weekend I went jogging around my residential area. Then my brother asked me why I was jogging? Then I said, “I need to cut down my weight so that I can pass out from [the programme]”. (008)*  *My [late] father [would] want me to be a better person. … I mean my dad’s side of my family, [in] his generation all have heart disease - heart attacks. So it’s a sign that I might also [be affected] … I don’t want to live the same way they did. (012)*  *During the first few weeks we had the jog and the routine, we experienced body aches every week. After the 10 weeks, we started to feel good when we ran. And like before the [graduation], I felt lighter, I felt good. (012)*  *I’m motivated to help my girl-friend and myself lose weight. We promise that by the end of this year we [are going to] lose about 10kg to 20kg. … That’s why I’ve been participating in more activities of late and trying to get into football and other sports. … Actually my goal is to get to size M or L before I get married. (013)*  *First thing - they [the military] need you to be fit, so that it is easy for you to go [into combat]. Second thing - look good, feel good. Make yourself light and when you want to work you have no difficulty. The third thing is they don’t want us to get high cholesterol, high blood [pressure]. (015)* | *[Back then] I didn’t really exercise. Usually after school I had to go to work. I [was] working part-time. On the weekend also, I worked part-time. (001)*  *I feel much healthier. I can do more. I can play more sports. (003)*  *I feel tired, I just run for a while. (003)*  *One guy from my batch. … I saw his photo once. He was also quite chubby. He now has become quite lean. I asked him “What do you do?” “Skip rope, do cardio, gym.” (007)*  *When you [are] exercising, you go around meeting different people. You will talk to them and make friends. (007)*  *At first, [the soccer team] didn’t want to take me as I was fat. I needed a sport, so I learnt to play takraw [South East Asian ball sport]. … By the time I lost weight, I was selected in the soccer team and then I played in both. (009)*  *After I gained weight, since last week, I’ve been doing [exercise] twice a day. (010)*  *I like to play soccer and volleyball. Usually, my stamina is not very good. Every time I participate in activities of reasonably high intensity, I have to take unnecessarily prolonged rests. My goal is to become fitter so that I can participate more in activities which interest me. (010)*  *They feel that they are okay with what they are. Some of them are attached [in a relationship]. The attached ones will say their girl-friend loves them the way they are. (010)*  *Nearing to the enlistment, I signed up to the gym for about 9 months. However, I didn’t really do weight loss training. I did more weights and muscle training. (014)*  *[In the gym] I will train both the upper body and lower body. It’s not about getting stronger. I just want to get fit. (016)* |
| Integrated Regulation | *They don’t give us much time to exercise. Every single day, we keep driving and driving. (002)*  *Basically a lot of IT (information technology) work that involves me sitting down a lot on the chair most of the time. (004)*  *It didn’t even strike me to exercise. My only physical activities were when I was working part-time as retail that involved me standing around. (004)*  *My bunk mate was posted to the infantry. He is maintaining his weight. He is a lot skinnier now. He is a lot stronger. For me and some of my friends in the transport vocation and storeman vocations, we exercise less, gaining more weight. (004)*  *Drivers do not have physical training sessions. They just drive all the way. (008)*  *I’m not really running very often recently also, because of army open house [a major event] and other duties. (011)*  *PT (Physical training) every morning is good. However, the issue is that PT every morning at seven plus is too early. We haven’t had breakfast so our body is still very weak. [When you] want to drive, you lose concentration already. (015)* | *Driver [vocation] is not stressful. Eight to five - just drive. Don’t need to exercise. (007)*  *Drivers are considered one of the relaxed vocations. It’s normal, you can ask anyone else. When you relax excessively, you gain weight. (009)*  *When I am off duty I can exercise on my own, I just have to write on the exercise board outside the office. They have this board. If you want to exercise by yourself, you just write you contact number and location and they will contact*  *you if they need you. (010)*  *But even [as a] storeman, I requested to join [exercise sessions]. That’s why I join them. If you don’t request [to exercise], you just relax. … I don’t think they actually requested to participate. We all exercise in [the residential programme] every day, that’s why we lose weight. But when you come out of the programme, you don’t exercise every day? No matter how little you eat, you will just gain back the weight. (010)*  *I started becoming overweight after around year 4 to 5 in primary school. … lack of exercise because, that’s when I started to get all the tutoring and no time to play outside. (014)*  *I have to keep fit, otherwise my [subordinates] will look down at me. (014)*  *I had to concentrate on my studies. I never went out of the house. All the way, I stayed in the house to study. There was no time to exercise. (016)* |
| Intrinsic Motivation | *I was into sports when I was young. But I think things changed when I grew up. (002)*  *I was only fit in primary 1 and primary 2. I used to win a lot of medals for sports day. After that, I let myself grow all the way until, secondary school. (004)*  *I used to play rugby but I stopped. (012)*  *I plan to go back to the gym again and swim, because I love to swim and I find I swimming allows me to lose the most amount of weight. (012)* | *Every week, I used to play [football] in the league. [I] used to play in the club. Last year I was supposed to play in the national football league. … In national service, you are only allowed to play only for your own division. (009)*  *I believe we should add in more sports, because the limitation of sports during the exercise is quite little. I believe no matter how fat … you can play sports without getting tired. … So if they implement sports, more sports might instead motivate them [other participants] even more. (009)*  *Usually, my stamina is not very good. Every time I participate in activities of reasonably high intensity, I have to take unnecessarily prolonged rests. My goal is to become fitter so that I can participate more in activities which interest me. (010)*  *I was actually having a pretty good time in [the programme]. It was fun. The exercise was tiring but after a while you get used to it, so it wasn’t too bad. (014)* |
